# Assessment of pulmonary surfactant in COVID-19 patients

**DOI:** 10.1186/s13054-020-03268-9

**Published:** 2020-09-07

**Authors:** Peter Schousboe, Lothar Wiese, Christian Heiring, Henrik Verder, Porntiva Poorisrisak, Povl Verder, Henning Bay Nielsen

**Affiliations:** 1 Department of Pediatrics, Holbaek University Hospital, Smedelundsgade 60, 4300 Holbaek, Denmark; 2 Department of Infectious Diseases, Zealand University Hospital Roskilde, Sygehusvej 10, 4000 Roskilde, Denmark; 3 Department of Neonatology, Neonatal and Pediatric Intensive Care Unit 5021 Rigshospitalet, University of Copenhagen, Copenhagen, Denmark; 4 Department of Anesthesia and Intensive Care, Zealand University Hospital Roskilde, Sygehusvej 10, 4000 Roskilde, Denmark

**Keywords:** COVID-19, ARDS, Pulmonary surfactant

The clinical presentation of coronavirus 2 (SARS-CoV-2) ranges from asymptomatic to severe respiratory failure, and correspondingly requirement for respiratory support ranging from varying levels of supplementary O_2_, to non-invasive and invasive ventilation. In a recent editorial by Gattinoni et al. [1], it is proposed that, based on different pathophysiology need for ventilator support, there are two groups of COVID-19 patients: one group that develops acute respiratory distress syndrome (ARDS) with low compliance, and another classified as non-ARDS with normal compliance and hypoxemia caused by a high level of intrapulmonary shunting. Furthermore, non-ARDS type COVID-19 pneumonia may transit to ARDS-type COVID-19 by self-inflicted or ventilator-induced lung injury.

We propose that the direct effects on pulmonary tissue by SARS-CoV-2 in COVID-19 pneumonia in ARDS COVID-19 patients resembles neonatal respiratory distress syndrome (NRDS), caused by surfactant deficiency.

## COVID-19 patients and pulmonary surfactant

SARS-CoV-2 enters and replicates in the alveolar type II cells impacting the production and turnover of pulmonary surfactants. This results in alveolar collapse and inflammation leading to increased capillary permeability, edema, and microvascular thrombosis; where the associated ARDS clinical picture closely resembles NRDS. Of importance, as ARDS progresses, vascular permeability increases and surfactant deactivates making the lung increasingly unstable [2]. Thus, clinical studies have provided promising data on the effect of surfactant treatment in patients with ARDS [3]. These pathophysiological findings have prompted several groups to investigate if surfactant administration could improve the COVID-19 patient outcome. Interventional trials are currently underway in the UK, USA, and Canada (ClinicalTrials.gov IDs: NCT04375735, NCT04362059, NCT04384731).

Treatment of NRDS with rescue surfactant and continuous positive airway pressure (CPAP), when compared to mechanical ventilation, results in reduced development of pulmonary fibrosis; a complication also reported in intubated COVID-19 patients. Antenatal corticosteroid treatment may also accelerate fetal lung maturation in the risk of preterm birth. A multicentre trial evaluates whether surfactant plus budesonide improves survival in NRDS [4]. In COVID-19 patients, dexamethasone seems to improve the outcome.

We suggest assessment of surfactant levels should be added to the evaluation of COVID-19 patients. A point-of-care test for fast measuring surfactant at birth in premature babies has been developed [5]. This method may be suitable for determining the surfactant in tracheal fluid obtained from critically ill adults. Knowledge of surfactant levels contributes to understanding pathophysiology in COVID-19 patients. Surfactant treatment may be considered included with other interventions for the treatment of COVID-19 induced respiratory failure.

## Data Availability

If necessary and considered needed by the journal, actions will be taken to obtain supporting data. If that is not the case, the subsection is considered not applicable.
